# Stereoselective pharmacokinetic and pharmacodynamic analysis of a CNS-active sulphamoylphenyl carbamate derivative

**DOI:** 10.1080/14756366.2019.1612887

**Published:** 2019-05-24

**Authors:** David Bibi, Bella Shusterman, Alessio Nocentini, Claudiu T. Supuran, Meir Bialer

**Affiliations:** a Faculty of Medicine, School of Pharmacy, Institute of Drug Research, The Hebrew University of Jerusalem, Jerusalem, Israel;; b Department of Neurofarba, University of Florence, Florence, Italy;; c Faculty of Medicine, School of Pharmacy, David R. Bloom Center for Pharmacy, The Hebrew University of Jerusalem, Jerusalem, Israel

**Keywords:** New antiepileptic drugs, 4-aminobenzenesulphonamides, CNS-active carbamates, carbonic anhydrase inhibition, pharmacokinetics

## Abstract

3-Methylpentyl(4-sulphamoylphenyl)carbamate (MSPC) came as the most potent compound out of a new series of carbamates composed of phenyl-ethanol or branched aliphatic alcohols, and 4-benzenesulphonamide-carbamic acid. In this study, the anticonvulsant activity and pharmacokinetics (PKs) of MSPC-two individual enantiomers were comparatively analysed in rats as well as their carbonic anhydrase (CA) inhibition. The anticonvulsant activity of MSPC enantiomers was evaluated at the rat-maximal electroshock (MES) test, and their CA inhibition evaluated. (R)-MSPC had a 29% higher clearance and consequently, a lower plasma exposure area under the curve (AUC) than (S)-MSPC and racemic-MSPC. Nevertheless, (R)-MSPC had a better brain permeability than its (S)-enantiomer with brain-to-plasma-(AUC)-ratio (BPR) of 2.07 ((R)-enantiomer), 1.85 (racemate), and 0.79 ((S)-enantiomer). As a whole body (*in vivo*) pharmacodynamic (PD) measure, MSPC-anticonvulsant maximal electroshock seizure (MES) activity was less enantioselective than MSPC-CA inhibition. The lack of significant differences between racemic-MSPC and its individual enantiomers suggest that their anticonvulsant activity might be due to multiple mechanisms of action.

## 1. Introduction

Despite the availability of more than 25 old and new antiepileptic drugs (AEDs), about 30% of the patients with epilepsy are not seizure-free with their current treatment. In addition, adverse events of existing AEDs restrict their use in certain segments of patients like women of child-bearing age. Therefore, there is an unmet clinical need to discover and develop novel chemical entities with a potential to become new effective and safer AEDs^1–3^.

As part of the attempts to develop new AEDs, a novel class of carbamates composed of phenyl-ethanol or branched aliphatic alcohols with 6–9 carbons and 4-benzenesulphonamide-carbamic acid was recently designed and evaluated for their anticonvulsant activity and carbonic anhydrase (CA) inhibition^4^.

**Figure 1.**
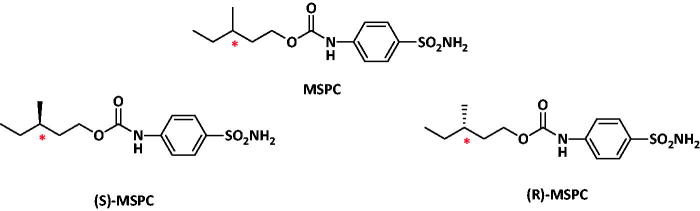
Structures of MSPC and its two individual enantiomers.

Out of the ten synthesised new carbamates the compound 3-methylpentyl(4-sulphamoylphenyl)carbamate (MSPC) came as the most potent compound with rat-MES-ED_50_ values of 13 mg/kg (i.p.) and 28 mg/kg (po)^4^. MSPC is a chiral compound with one stereogenic centre and thus, racemic-MSPC is composed of two individual enantiomers depicted in Figure 1. Enantiomers possess the same chemical formula, but differ in their three-dimensional arrangement around the stereogenic carbon and exist as two non-super imposable froms^5^ ^,^ ^6^.

The issue of drug chirality is a major theme in the design, discovery, and development of new active pharmaceutical entities due to the understanding of the role of stereospecificity and molecular recognition in drugs activity. Thus, most of the new chiral drugs reaching the market are single enantiomers, rather than racemic mixtures^7^. Enantiomers are often readily distinguished by biological systems and may have different pharmacokinetic (PK) or pharmacodynamic (PD) properties in their wanted (designated) indication or unwanted effects^5^ ^,^ ^6^.

In this study, we assessed the effect of enantioselectivity and stereospecificity of the two enantiomers of MSPC, a new molecule containing carbamate and sulphonamide, two moieties incorporated in the olds AEDs (e.g. acetazolamide felbamate and zonisamide) as well as new AEDs in the pipeline (e.g. cenobamate and padsevonil)^2^ ^,^ ^3^. The experience learnt from felbamate and carisbamate coupled with the results of the currently ongoing clinical studies with cenobamate and padsevonil emphasises the desire to develop new AEDs containing sulphamoylphenyl and/or carbamate moieties in their chemical structure. Thus, in this study, we synthesised and comparatively analysed the anticonvulsant activity of MSPC two individual enantiomers as well as their CA inhibition and PK in rats.

## 2. Materials and methods

### 2.1. Chemistry

All the solvents were of analytical grade or high-performance liquid chromatography (HPLC) grade and were purchased from Sigma-Aldrich, St. Louis, MO. The synthesis and chemical structures of racemic-MSPC was previously described^4^.

### 2.2. General procedure for the synthesis of (S)- or (R)-MSPC

A solution of (S)-methylvaleric acid (for the synthesis of (S)-MSPC) or (R)-methylvaleric acid (for the synthesis of (R)-MSPC) (1 equiv) in dry tetrahydrofuran (THF) (10 ml) was added dropwise to a stirred solution of lithium aluminium hydride (1.2 equiv) in dry THF (20 ml). The reaction mixture was allowed to stir for 2 h and then quenched with water and extracted with ethyl ether (3 × 30 ml). The organic layer was dried over sodium sulphate, filtered, and evaporated to yield 80% oily product. The coupling product was conjugated with sulfanilamide according to a previously published method^4^. MSPC enantiomers and their purity were assessed by ^1^H NMR, HPLC and elemental analysis and the results obtained were as follows.

#### 2.2.1 (R)- or (S)-3-methylpentyl(4-sulphamoylphenyl)carbamate (MSPC)

^1^H NMR (300 MHz, DMSO-d_6_) δ 10.00 (s, 1H), 7.70 (d, *J* = 8.8 Hz, 2H), 7.59 (d, *J* = 8.7 Hz, 2H), 7.20 (s, 1H), 4.18–4.07 (m, 2H), 1.73–1.56 (m, 1H), 1.11–1.21 (m, 4H), and 0.85 (m, 6H). Calculated for C_13_H_20_N_2_O_4_S: C, 51.98; H, 6.71; N, 9.33; S, 10.68. Found: C, 52.12; H, 6.54; N, 9.35; S, 10.77.

### 2.3. Pharmacokinetics studies

The pharmacokinetics (PK) of racemic-MSPC and its two individual enantiomers were studied following i.p. (80 mg/kg) to rats and their major PK parameters were estimated. MSPC has low water solubility, and consequently, it was administered to rats in multisol of propylene glycol, alcohol, and water for injection at a ratio of 8:1:1.

The study details were as described previously^8^ and plasma and brain levels of racemic-MSPC and it individual enantiomers were monitored at: 20, 40, 60, 90, 120, 160, 180, 200, 220, 260, 320, and 360 min after dosing.

### 2.4. Analysis of MSPC and its two individual enantiomers in plasma and brain

Plasma and brain concentrations of each compound were quantified by an HPLC assay. The HPLC analysis was performed on a system (2695 Separation Module; Waters, Milford, MA) with a photodiode array UV detector (2996 PDA Detector; Waters, Milford, MA). Conditioned as follows: Kinetex, 5 u EVO C18 100 A, 150*4.6 mm column (Phenomenex^®^, Torrance, CA). Linear gradients (5–95% acetonitrile content) with H_2_O (0.1% formic acid) and acetonitrile were used as the eluents with flow rate of 1 ml/min at 20 °C. The compounds and the internal standard were detected at 250 nm. Plasma and brain concentrations of MSPC and its two individual enantiomers were quantified by assay that its extraction method was previously described^8^.

### 2.5. Analysis of MSPC in urine

MSPC urine concentrations were quantified by the same HPLC assay as its plasma assay.

### 2.6. Calculation of pharmacokinetic (PK) parameters

The PK parameters of each compound were calculated by non-compartmental analysis based on statistical moment theory using the PK software Phoenix Winnonlin Tripos L.P. (Pharsight Co., Mountain View, CA) as previously described^9^. MSPC amount excreted unchanged in the urine was calculated by multiplying its urine concertino by the excreted urine volume for up to 6 h after dosing.

### 2.7. Anticonvulsant activity of MSPC and its individual enantiomers

The experiments at the MES model were done in male Sprague-Dawley rats (8 rats per dose) weighing 100−120 g (Charles River Laboratories, Wilmington, MA) as previously described^9–11^.

### 2.8. Carbonic anhydrase inhibition of MSPC and its individual enantiomers

The CA inhibition of MSPC and its individual enantiomers were assessed by a stopped flow CO_2_ hydrase assay as previously described^12–15^.

## 3. Results

The synthesis of (R)- and (S)-MSPC enantiomers was as follows:


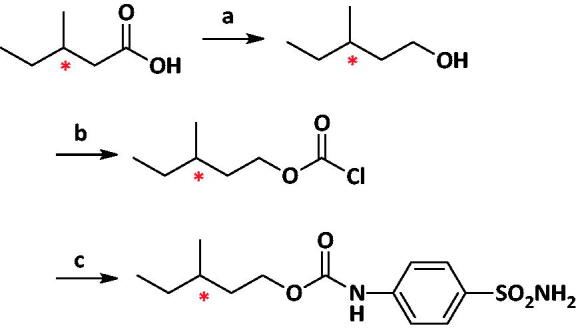


Conditions and reagents: (a) Lithium aluminium hydride, Ethyl ether, 0 °C 4 h (b) pyridine, triphosgene, DCM, RT, 2 h; (c) 4-aminobenzenesulphonamide, THF, RT 12 h.

**Figure 2.**
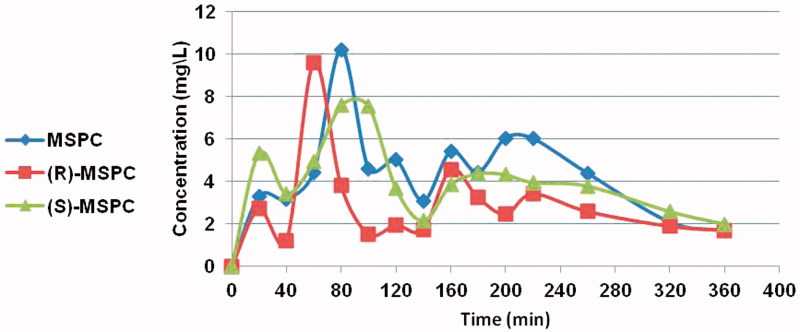
MSPC, (S)-MSPC, and (R)-MSPC plasma concentrations after i.p. (80 mg/kg) administration to rats.

**Figure 3.**
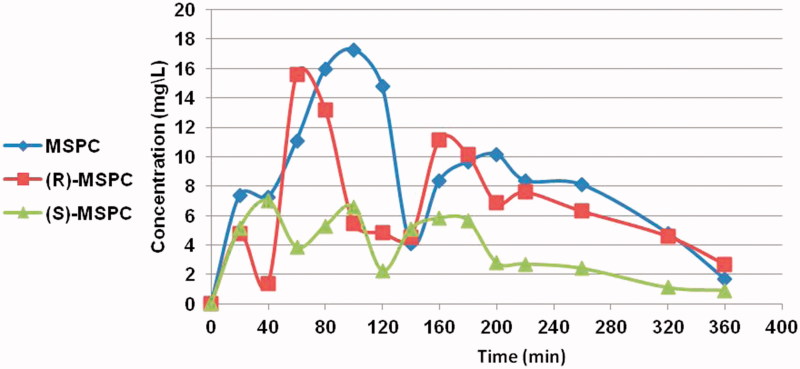
MSPC, (S)-MSPC, and (R)-MSPC brain concentrations after i.p. (80 mg/kg) administration to rats.

The major PK parameters of MSPC (racemate) and its individual enantiomers are depicted in Tables 1 and 2. The mean MSPC plasma and brain concentration *versus* time plots are presented in Figures 2 and 3, respectively. Less than 0.1% of MSCP dose was excreted unchanged in urine.

**Table 1.** Mean PK parameters of the MPSC (racemate) and its two individual enantiomers calculated from plasma levels following i.p. (80 mg/kg dose) administration to rats.

PK parameters	Racemate	(S)-enantiomer	(R)-enantiomer
t_1/2_ (h)	2.1	2.3	2.5
CL/F (l h^−1^ kg^−1^)	0.7	0.7	0.9
Vss/F (l/kg^−1^)	2.2	2.3	3.2
AUC (mg l^−1^ h^−1^ )	29	29	23
C_max_ (mg/l)	10	7.6	10
t_max_ (h)	1.3	1.3	1.0

**Table 2.** Mean PK parameters of the MPSC (racemate) and its two individual enantiomers calculated from brain levels following i.p. (80 mg/kg dose) administration to rats.

PK parameters	Racemate	(S)-enantiomer	(R)-enantiomer
t_1/2_ (h)	1.9	1.7	2.3
CL/F (l h^−1^ kg^−1^)	0.4	0.9	0.4
Vss/F (l/kg^−1^)	1.0	2.2	1.4
AUC (mg l^−1^ h^−1^ )	53	23	47
C_max_ (mg/l)	18	7.0	16
t_max_ (h)	1.6	0.6	1.0

The brain-to-plasma-(AUC)-ratio (BPR) of MSPC and its two individual enantiomers were as follows:

$$\text{Racemate}={\text{AUC}}_{\text{brain}}/{\text{AUC}}_{\text{plasma}}=52.8/28.5\;=\;1.85.$$

$$\left(R\right)-\text{enantiomer}={\text{AUC}}_{\text{brain}}/{\text{AUC}}_{\text{plasma}}=46.9/24.8\;=\;2.07.$$

$$\left(S\right)-\text{enantiomer}={\text{AUC}}_{\text{brain}}/{\text{AUC}}_{\text{plasma}}=22.8/29\;=\;0.79.$$

The rat-MES (po)-ED_50_ values (and their 95% confidence interval - 95% CI) of racemic-, (S)- and (R)-MSPC were: 28 mg/kg (18–25 mg/kg), 24 mg/kg (17–29 mg/kg), and 30 mg/kg (22–37 mg/kg), respectively.

Table 3 shows the inhibition constant (Ki) of racemic-MSPC and its two individual enantiomers against four human carbonic anhydrase (hCA) isoforms. The compounds’ potency varied from nanomolar to hundreds of nanomolars, depending on the hCA isoform. MSPC was not a potent inhibitor of CAs, CAs IV, and VII, but inhibited of CAs I and II. (R)-MSPC was 3.8 and 6.1 a more effective inhibitor than (S)-MSPC, against hCAs I and II, respectively.

**Table 3.** Inhibition data of human CA isoforms hCA I, II, IV, and VII with MPSC as racemate and single enantiomers and the standard sulphonamide inhibitor acetazolamide (AAZ).

K_I_(nM)*				
Compound	hCAI	hCA II	hCA IV	hCA VII
MSPC (racemate)	77.0	7.6	750	351
(S)-MSPC	130	20.3	593	744
(R)-MSPC	34.1	3.3	810	170
AAZ	250	12	74	2.5

*Mean from three different assays, by a stopped flow technique (errors were in the range of ±10% of the reported values).

## 4. Discussion

Among our current antiepileptic armamentarium as well as among the AEDs in development there are drugs containing carbamate or sulphonamide moieties in their chemical structure. Felbamate is a carbamate that exhibits a broad anticonvulsant activity and has been on the market since 1993. However, currently, it is seldom used due to the fatal aplastic anaemia and hepatotoxicity that is associated with its therapy^16^ ^,^ ^17^. Two additional carbamates, carisbamate, and cenobamate completed phase III clinical trials and their new drug application (NDA) were submitted to the Food and Drug Administration (FDA). Carisbamate regulatory application was withdrawn in 2010, due to lack of consistent efficacy across a clinically-relevant dose range^18–19^. Two cenobamate well-controlled studies demonstrated statistically significant reduction in seizure frequency as well as high responder rates, including seizure freedom, in patients with uncontrolled partial seizures treated with cenobamate for up to 18 weeks^18–22^. Due to three reported cases of drug reaction with eosinophilia and systemic symptoms (DRESS) in patients exposed to cenobamate, a large Phase III open-label study took place to evaluate the long-term safety of cenobamate when using a lower starting dose (<100 mg) and slower titration rate in order to mitigate the serious coetaneous reactions (e.g. DRESS)^22^. Subsequently, cenobamate-NDA submission was accepted by the FDA on 4/2/2019 and its Prescription Drug User Fee Act (PDUFA) date is set for 21/11/2019^23^.

The human central nervous system (CNS) is among the tissues/organs having the highest number of CA isoforms, among which CA I, II, III, IV, VA, VII, XII, and XIV^24^. Given the multitude of CA isoforms in the CNS, their inhibition was exploited already in the 1970s as a source for new AEDs. The mechanisms by which CA isoforms possess antiepileptic activity is rather complex and there is no definitive consensus among researchers about this issue^24^.

Thus, CA inhibitors (e.g. acetazolamide and zonisamide) are among the AEDs in our current therapeutic arsenal. Acetazolamide (1953) and zonisamide (Japan-1989; USA-2000) are marketed AEDs containing sulphonamide in their chemical structure^25^ ^,^ ^26^. Padsevonil is a new AED in development designed by UCB Pharma that contains benzenesulphonamide in its chemical structure and inhibits seizure activity *via* presynaptic modulation of the SV2 isoforms as well as postsynaptic enhancement of GABA-mediated inhibition^3^ ^,^ ^27^.

Researchers at Sumimoto Dainippon Pharma are also currently developing new AEDs based on the scaffold of benzenesulphonamide such as 2'-fluoro-N-methyl-[1,1'-biphenyl]-2-sulphonamide and other N-alkyl-[1,1'-biphenyl]-2-sulphonamide derivatives^28^ ^,^ ^29^. In addition, a few groups designed and evaluated substituted 4-aminobenzene sulphonamides, relying on Lipinski rule or hCA I inhibitors as potential novels AEDs^30^ ^,^ ^31^.

MSPC was the most potent compound of a series of sulphamoylphenyl or alkyl carbamate deraivtives^4^. As a chiral molecule, MSPC has to be developed as a single individual enantiomer^7^ ^,^ ^32^ ^,^ ^33^. The current stereoselective PK and PD analysis showed that in spite of its lower (78%) plasma exposure (compared to (S)-MSPC), (R)-MSPC had a 2.6 higher BPR than its enantiomer. Nevertheless, the rat-MES-ED_50_ values of MSPC and its individual enantiomers were similar, ranging from 24 to 30 mg/kg. Thus, both MSPC-two individual enantiomers might be candidates for further evaluation as potential new AEDs.

This research also demonstrated that a whole body (*in vivo*) PD measure such as MSPC-anticonvulsant activity (MES) was less enantioselective than MSPC-specific PD measures CA inhibition or its primary PK parameter clearance that was mainly metabolic. (R)-MSPC better BPR might be also due to the fact that it is a more potent inhibitor (compared to the (S)-enantiomer) of the brain-abundant CA isoforms CA I and CA II. The lack of significant differences between MSPC and its individual stereoisomers suggest that their anticonvulsant (MES) activity might be due to multiple mechanisms of action.
